# Curcumin Inhibits Tumor Growth and Angiogenesis in an Orthotopic Mouse Model of Human Pancreatic Cancer

**DOI:** 10.1155/2013/810423

**Published:** 2013-11-10

**Authors:** Sabrina Bimonte, Antonio Barbieri, Giuseppe Palma, Antonio Luciano, Domenica Rea, Claudio Arra

**Affiliations:** ^1^Istituto Nazionale per lo Studio e la Cura dei Tumori “Fondazione G. Pascale”, IRCCS, Via Mariano Semmola, 80131 Naples, Italy; ^2^Istituto di Endocrinologia e Oncologia Sperimentale del Consiglio Nazionale delle Ricerche, Dipartimento di Biologia e Patologia Cellulare e Molecolare “L. Califano”, Università degli Studi di Napoli “Federico II”, Via Pansini 5, 80131 Napoli, Italy

## Abstract

Pancreatic cancer is a malignant neoplasm originating from transformed cells arising in tissues forming the pancreas. The best chemotherapeutic agent used to treat pancreatic cancer is the gemcitabine. However, gemcitabine treatment is associated with many side effects. Thus novel strategies involving less toxic agents for treatment of pancreatic cancer are necessary. Curcumin is one such agent that inhibits the proliferation and angiogenesis of a wide variety of tumor cells, through the modulation of many cell signalling pathways. In this study, we investigated whether curcumin plays antitumor effects in MIA PaCa-2 cells. *In vitro* studies showed that curcumin inhibits the proliferation and enhances apoptosis of MIA PaCa-2 cells. To test whether the antitumor activity of curcumin is also observed *in vivo*, we generated an orthotopic mouse model of pancreatic cancer by injection of MIA PaCa-2 cells in nude mice. We placed mice on diet containing curcumin at 0.6% for 6 weeks. In these treated mice tumors were smaller with respect to controls and showed a downregulation of the transcription nuclear factor NF-**κ**B and NF-**κ**B-regulated gene products. Overall, our data indicate that curcumin has a great potential in treatment of human pancreatic cancer through the modulation of NF-**κ**B pathway.

## 1. Introduction

Pancreatic cancer is a malignant neoplasm originating from transformed cells arising in tissues forming the pancreas, and it is now the fourth most common cause of cancer-related deaths in the United States and the eighth worldwide [1]. This pathology leads to an aggressive local invasion and early metastases, and is poorly responsive to treatment with chemotherapy [2]. At present, gemcitabine is the best chemotherapeutic agent available for treatment of pancreatic cancer. However, it is noted that patients with this cancer treated with gemcitabine, showed several side effects and developed drug resistance over time [3]. In order to bypass these problems, novel strategies involving less toxic agents that can sensitize pancreatic cancer cells to chemotherapy are necessary. Curcumin, a component of the spice turmeric (*Curcuma longa*), is one such agent and is not toxic to humans [4]. It has been used for thousands years in Orient as a healing agent for variety of illnesses. Many studies provided evidence that curcumin is an agent with strong inflammatory properties and strong therapeutic potential against many variety of cancer. Curcumin inhibits the cell survival, proliferation, and angiogenesis of a wide variety of tumor cells, through the modulation of various cell signalling pathways, which involve transcription factor, protein kinases, growth factor, and other enzymes. In particular, it has been demonstrated that curcumin modulates the activation of the transcription factor nuclear factor-*κ*B (NF-*κ*B) which, in turn, plays a role in the growth and chemoresistance of pancreatic cancer [5, 6]. It has been showed that NF-*κ*B promotes pancreatic growth by inhibiting apoptosis [7–9]. Additionally it has been demonstrated that NF-*κ*B plays also several roles in angiogenesis [10], migration, and invasion of pancreatic cells [11]. Curcumin has also been shown to suppress angiogenesis *in vivo* [12–14]. *In vitro* and *in vivo* studies have already demonstrated that curcumin inhibits the growth of human pancreatic cancer cells [15, 16]. Previously, it has been demonstrated that curcumin can potentiates the antitumor activity of gemcitabine in pancreatic cells by downregulating NF-*κ*B-regulated gene products [17]. 

In this study, we assessed the antitumor activities of curcumin in human pancreatic cancer cell line and MIA PaCa-2 cells by *in vitro* and *in vivo* experiments. *In vitro* data allowed us to demonstrate that curcumin, inhibited the proliferation and enhanced the apoptosis of MIA PaCa-2 cells. *In vivo*, by generation of an orthotopic mouse model of pancreatic cancer, we showed that tumours from mice injected with MIA PaCa-2 cells and placed on diet containing curcumin at 0.6% for 6 weeks were smaller than those observed in controls. We also showed a down regulation of the NF-*κ*B-regulated gene products (cyclin D, VEGF, MMP-9, and IKK*α*/*β*). Overall, our data indicate that curcumin has a great potential in treatment of human pancreatic cancer, through the modulation of NF-*κ*B pathway.

## 2. Materials and Methods

### 2.1. Materials

Curcumin, (E,E)-1,7-bis (4-Hydroxy-3-methoxyphenyl)-1,6-heptadiene-3,5-dione, Diferuloylmethane, Diferulylmethane, Natural Yellow 3 used for *in vitro* experiments, was obtained from Sigma Aldrich (Piscataway, NJ). The following polyclonal antibodies cyclin D1, MMP-9 and monoclonal antibodies against VEGF were obtained from Santa Cruz Biotechnology (Santa Cruz, CA). Anti-IKK*α* and anti-IKK*β* antibodies were kindly provided by Imgenex (San Diego, CA). The liquid DAB+ Substrate Chromogen System-HRP used for immunocytochemistry was obtained from DakoCytomation (Carpinteria, CA). Penicillin, streptomycin, RPMI 1640, and fetal bovine serum (FBS) were obtained from Invitrogen (Grand Island, NY). Tris, glycine, NaCl, SDS, and bovine serum albumin (BSA) were obtained from Sigma Chemical (St. Louis, MO). Complete feed for mice with curcumin 0.6% (AIN-93G) was purchased by Mucedola (Settimo Milanese, Italy). 

### 2.2. Cell Lines

The pancreatic cancer cell line MIA PaCa-2 transfected with red fluorescent protein (RPF) and MPanc-96 cells was a kind gift from Professor Turco (University of Fisciano, Italy). Panc-1 cells were obtained from the American Type Culture Collection (Manassas, VA). All cell lines were cultured in RPMI 1640 supplemented with 10% FBS, 100 units/mL penicillin, and 100 *μ*g/mL streptomycin. 

### 2.3. Proliferation Assay

The effect of curcumin on cell proliferation was determined by using TACS 3-(4,5-dimethylthiazol-2-yl)-2,5-diphenyltetrazolium bromide (MTT) cell proliferation assay (Trevigen, Githersburg). The cells (2,000 per well) were incubated with or without curcumin, in triplicate in a 96-well plate and then incubated for 2, 4 and 6 days at 37°C. A MTT solution was added to each well and incubated for 2 h at 37°C. An extraction buffer (20% SDS and 50% dimethylformamide) was added, and the cells were incubated overnight at 37°C. The absorbance of the cell suspension was measured at 570 nm using a microplate reader (DAS Technologies, Chantilly, VA). This experiment was repeated twice, and the statistical analysis was performed to obtain the final values.

### 2.4. Wound-Healing Assay

MIA PaCa-2 cells were seeded at the density of 40 × 10^3^ cells per well into a 6-multiwell plate and cultured in RPMI 1640 medium supplemented with 1% FBS. At the time of confluence, cells were incubated in the absence or presence of curcumin (50 *μ*M) for 48 h after a slit made horizontally with a white tip at the center of each confluent well. Cell invasion on the slit of the confluent well, was assessed at 0, 24, and 48 hours in each condition by light microscopy.

### 2.5. *In Vitro* Apoptosis Assay by Flow Cytometry

Cells were washed and suspended in 0.5 mL of PBS, and 1 AL/mL YO-PRO-1, and propidium iodide was added. Cells were incubated for 30 min on ice and analyzed by flow cytometry (FACScan, Becton Dickinson, Franklin Lakes, NJ) by measurements of fluorescence emission at 530 and 575 nm. 

The apoptotic cells were stained with the green fluorescent dye YO-PRO-1 while necrotic cells were stained with propidium iodide. The apoptotic fraction was obtained by dividing the number of apoptotic cells by the total number of cells (minimum of 10^4^ cells). Data were analyzed using Cell Quest software (Becton Dickinson). All data were reproduced at least thrice in independent experiments. 

### 2.6. Transfection of Small Interfering RNA

MIA PaCa cells in 96-well plates were grown to 50% confluence and transfected with double-stranded siRNA for relA/p65 (form of NF-*κ*B) target sequence (Sense 5′ CCAUCAACUAUGAUGAGUU dTdT 3′, Antisense 3′ dGdTGGUAGUUGAUACUACUCAA 5′) or with a siRNA nonspecific control (Ambion, Austin, TX, cat n.461) in serum-free medium without antibiotic supplements using Hiperfect Transfection Reagent (Qiagen, Inc.). Cells were incubated under these conditions for 48 h and silencing was then confirmed by western blotting analysis.

### 2.7. NF-*κ*B Activation in Cell and Tumor Samples

To assess NF-*κ*B activation, we performed electrophoresis mobility shift assays (EMSA) on isolated nuclei from pancreatic cancer tumor and cell samples as described previously [17, 18]. Briefly, nuclear extracts prepared from pancreatic cancer cells (1 × 10^6^/mL) and tumor samples were incubated with 32P-end-labeled 45-mer double-stranded NF-*κ*B oligonucleotide (4 *μ*g of protein with 16 fmol of DNA) from the HIV long terminal repeat (5′-TTGTTACAAGGGACTTTCCGCTGGGGACTTTCCAGGGAGGCGTGG-3′; indicates NF-*κ*B-binding sites) for 15 min at 37°C. The resulting DNA-protein complex was separated from free oligonucleotide on 6.6% native polyacrylamide gels. A double-stranded mutant oligonucleotide (5′-TTGTTACAACTCACTTTCCGCTGCTCACTTTCCAGGGAGGCGTGG-3′) was used to examine the specificity of binding of NF-*κ*B to the DNA. The dried gels were visualized, and radioactive bands were quantitated using Phosphor Imager (Molecular Dynamics, Sunnyvale, CA) using Image Quant software. 

### 2.8. Mice

Sixteen eight-week-old female upstream to Foxn1 mice were purchased by Harlan, San Pietro al Natisone, and (Italy). Mice were housed five for cage in the standard mice Plexiglas cages and maintained on a 12 h light : 12 h dark cycle (lights on at 7.00 am) in a temperature-controlled room (22 ± 2°C) and with food and water ad libitum at all times. The experimental protocols were in compliance with the European Communities Council directive (86/609/EEC).

### 2.9. Generation of Orthotopic Mouse Model of Pancreatic Cancer and Experimental Protocol

RFP-transfected MIA PaCa-2 cells were harvested from subconfluent cultures after a brief exposure to 0.25% trypsin. Trypsinization was stopped with medium containing 10% FBS. The cells were washed once in serum-free medium and suspended in PBS. Only suspensions consisting of single cells, with >90% viability, were used for the injections. A total of 16 female Foxn1 nu/nu mice were used in this experiment and maintained in a barrier facility on HEPA-filtered racks. Animals were individually identified using numbered ear tags. All experiments were conducted in a biological laminar flow hood, and all surgical procedures were conducted with strict adherence to aseptic technique. The mice were anesthetized with avertin solution injected intraperitoneally according to their weight. Then mice were prepped with 10% povidone-iodine on the left flank and draped in a sterile fashion. A longitudinal median laparotomy with a xiphopubic incision was made, and the tail of the pancreas is exteriorized gently. A suspension of 5 × 10^5^ MIA PaCa 2-RFP cells in 25 *μ*L of PBS 1X/mouse was injected into the tail of the pancreas by using a 29-gauge needle and a calibrated push button-controlled dispensing device (Becton Dickinson, Franklin Lakes, NJ). To prevent leakage, the injection point was dubbed with sterile cotton. Once haemostasis was confirmed, the tail of the pancreas was returned into the abdomen and the abdominal wound was closed in a single layer using interrupted 5-0 silk sutures (US Surgical, Norwalk, CT) and skin staples. After 2 week of implantation, mice were randomized into the following treatment groups (*n* = 6) on the basis of fluorescent area measured by MacroFluo imaging: (a) untreated mice placed in normal died; (b) curcumin treated mice placed in diet containing curcumin at 0.6%. Tumor volumes were monitored once a week by using MacroFluo and LAS V3.7 software Leica Microsystems s.r.l. (Switzerland, Ltd). Before imaging, mice were anesthetized with Avertin solution. At each imaging time point, the real-time determination of tumor burden was performed by quantifying fluorescent surface area. Graph depicts tumor area means at 35 weeks after tumor cells injection. Therapy was continued for 4 weeks and animals were sacrificed 2 weeks later. Primary tumors in the pancreas were excised, and the final tumor volume was measured as *V* = 2/3*πr*3, where *r* is the mean of the three dimensions (length, width, and depth). Statistical analysis was performed to detect the final tumor volumes (paired *t*-test) by Graph Pad Prisme 5.0. Half of the tumor tissue was formalin fixed and paraffin embedded for immunohistochemistry and routine H&E staining. The other half was snap frozen in liquid nitrogen and stored at −80°C. H&E staining confirmed the presence of tumor(s) in each pancreas. 

### 2.10. Preparation of Nuclear Extract from Tumor Samples

Pancreatic tumor tissues (75–100 mg/mouse) from control and experimental mice were prepared as previously described [17]. The supernatant (nuclear extract) was collected and stored at −70°C until use. Protein concentration was determined by the Bradford protein assay with BSA as the standard. 

### 2.11. Immunohistochemical Analysis for VEGF and COX-2 in Tumor Tissue

Pancreatic cancer tumor samples from control and treated mice were embedded in paraffin and fixed with paraformaldehyde. After being washed in PBS, the slides were blocked with protein block solution (DakoCytomation) for 20 min and then incubated overnight with mouse monoclonal antihuman VEGF and anti-COX-2 antibodies (1 : 60 and 1 : 80, resp.). After the incubation, the slides were washed and then incubated with biotinylated link universal antiserum followed by horseradish peroxidase-streptavidin conjugate (LSAB + kit). The slides were rinsed, and color was developed using 3,3′-diaminobenzidine hydrochloride as a chromogen. Finally, sections were rinsed in distilled water, counterstained with haematoxylin and mounted with DPX mounting medium for evaluation. Pictures were captured with a Photometrics CoolSNAP color camera (Nikon, Lewisville, TX) and MetaMorph version 4.6.5 software (Universal Imaging, Downingtown, PA). 

### 2.12. Western Blot Analysis

Pancreatic tumor tissues (75–100 mg/mouse) from control and experimental mice were minced and incubated with ice for 1 h in 0.5 mL of ice-cold Lysis Buffer (10 mM Tris, ph 8.0, 130 mM Nacl, 1% Triton X-100, 10 mM NaF, 10 mM sodium phosphate, 10 mM sodium pyrophosphate, 2 *μ*g/mL aprotinin, 2 *μ*g/mL leupeptin, and 2 *μ*g/mL pepstatin). The minced tissue was Homogenized using a Dounce homogenizer and centrifuged at 16,000 ×g at 4°C for 10 min. Western blotting analysis was performed as described previously [17]. *β*-Actin was used as loading control. 

## 3. Results

### 3.1. Curcumin Inhibits Proliferation and Enhanced Apoptosis of Pancreatic Cancer Cells *In Vitro*

**Figure 1:**
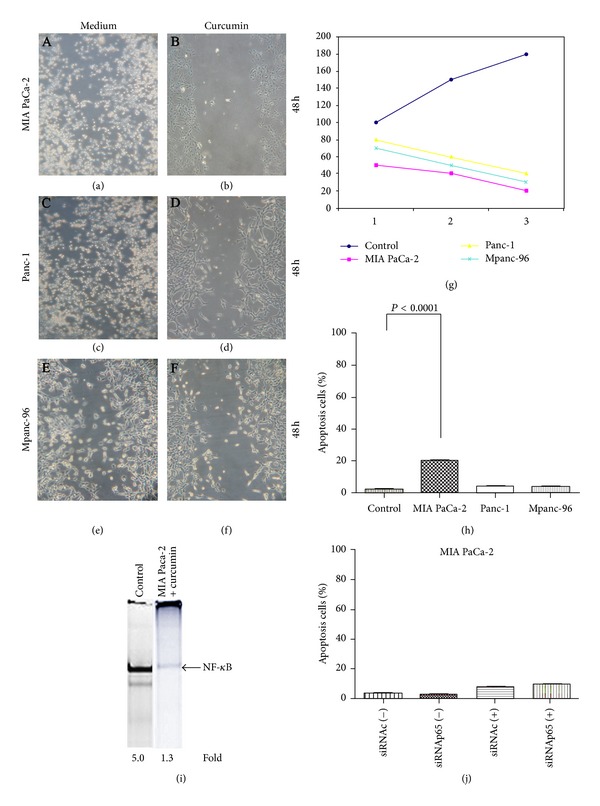
Curcumin inhibits proliferation and enhances apoptosis in MIA PaCa-2 cells. (a)–(f) Wound assay results show suppression of MIA PaCa-2 cell proliferation induced by curcumin at 48 h (b) With respect to controls. The results are the mean of total invaded areas of three distinct sections on the slide per 48 h. (*P* = 0.02, Mann-Whitney *U* test). (g) MTT assay results show a suppression of proliferation in pancreatic cancer cells treated with curcumin respect to control cells. Data are representative of two independent experiments (*P* value <0.05). (h) *In vitro* apoptosis assay by flow cytometry indicated that curcumin enhances apoptosis in MIA PaCa-2 cells. (i) EMSA results showes that curcumin suppresses activation of NF-*κ*B in MIA PACA-2 cells. (j) *In vitro* apoptosis assay by flow cytometry indicates that silencing of NF-*κ*B using siRNA *κ*B is not able to potentiate the apoptotic effects of curcumin on MIA PaCa-2 cells.

We first determined whether curcumin inhibits the proliferation of human pancreatic cancer cells by performing *in vitro* assays on MPanc-96, Panc-1, and MIA PaCa-2 cells. Wound healing assay demonstrated that curcumin (50 *μ*M), inhibits the proliferation of pancreatic cancer cell lines at 48 h (Figures 1(a)–1(f)). These results were also confirmed by MTT assay (Figure 1(g)). In order to asses if curcumin enhances the apoptosis in pancreatic cancer cells, we also performed *in vitro* apoptosis assay by flow cytometry. Our results showed that the percentage of apoptosis of MIA PaCa-2 cells treated with curcumin was higher with respect to controls and to MPanc-96 and Panc-1 cells (Figure 1(h)). Since the effects of curcumin were more evident in MIA PaCa-2 cells, we selected this pancreatic cancer cell line for further experiments. It has been demonstrated that curcumin modulates the activation of the transcription factor nuclear factor-*κ*B (NF-*κ*B) for this reason, we performed a nuclear NF-*κ*B DNA binding assay on MIA PaCA-2 cells treated with curcumin and controls, and we demonstrated that curcumin downregulated NF-*κ*B activation in MIA PaCa-2 cells (Figure 1(i)). To test the hypothesis that inhibition of NF-*κ*B would increase the sensitivity of MIA PaCa-2 cells to curcumin-mediated apoptosis, we used small interfering RNA (siRNA) to knock down p65/relA (a form of NF-*κ*B). Silencing was confirmed by reporter assays as well as western blotting (data not shown). Next, we transiently silenced expression of p65/relA in MIA PaCa-2 cells and examined the effects on the apoptosis alone and in presence of curcumin. We demonstrated that silencing of NF-*κ*B is not able to potentiate the apoptotic effects of curcumin on MIA PaCa-2 cells (Figure 1(j)). 

### 3.2. Curcumin Inhibits the Tumor Growth in Orthotopic Mouse Model of Pancreatic Cancer

**Figure 2:**
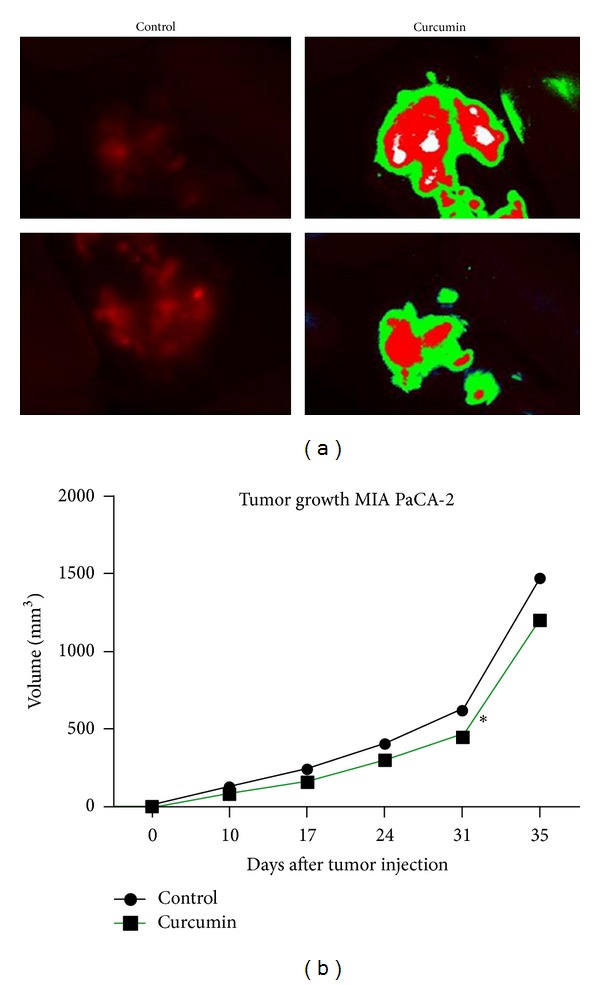
Curcumin inhibits the tumor growth in orthotopic mouse model of pancreatic cancer. (a) MacroFluo images of fluorescent analysis tumor area images control and treated mice. (b) Measurements of fluorescence per second depicting tumor volume at different time points using Macrofluo images, showed that the final tumor volumes on day 35 after the start of treatment is significantly decreased in the curcumin group compared with control (*P* = 0.00393).

In order to study the role curcumin on the tumor growth *in vivo*, we generated a mouse model of pancreatic cancer by injection of MIA PaCa-2-RFP cells in the pancreas of nude mice. Based on the MacroFluo images, 2 weeks later of cell injection, the mice were randomized into two groups: control group (normal diet) and curcumin group (feed added with 0.6% curcumin). The treatment with feed added with 0.6% curcumin was started after the tumor cell implantation and continued for 6 weeks. We also monitored the body weight of mice twice a week until the end of treatment. No difference was observed between the body weight of two groups of animals, indicating that mice eat normally complete fed with curcumin. Mice were sacrificed at the end of treatment. The images were performed on days 10, 17, 24, and 31 after the start of treatment (Figure 2(a)). The bioluminescence imaging results indicated a gradual increase in tumor volume in the curcumin group compared with treatment groups. The final tumor volumes on day 35 after the start of treatment showed a significant decrease in the curcumin group compared with control (Figure 2(b)). These data were also confirmed on tumor measurements performed at the end of the experiment at autopsy using digital Caliper and calculated using the formula *V* = 2/3*πr*3 (*n* = 6) (data not shown). 

### 3.3. Curcumin Inhibits NF-*κ*B Activation and Down-Regulates NF-*κ*B-Regulated Gene Products in Orthotopic Pancreatic Tumors

**Figure 3:**
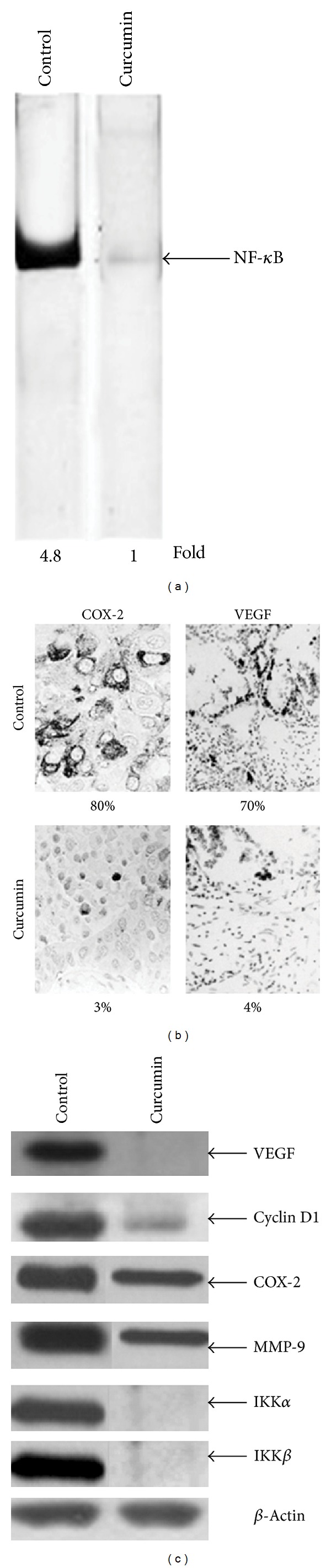
Curcumin inhibits NF-*κ*B activation and downregulates NF-*κ*B-regulated gene products in orthotopic pancreatic tumors. (a) EMSA assay performed on orthotopic tumor tissue samples showed the inhibition of NF-*κ*B by curcumin. (b) Immunohistochemical analysis for nuclear COX-2 and VEGF showed the inhibition of COX-2 and VEGF expression in curcumin-treated group, compared to controls (Figure 3(b), lower panels). Percentages indicate the positive staining for the given biomarker. (c) Western blot showing that curcumin inhibits the expression of NF-*κ*B-dependent gene products VEGF, cyclin D1, MMP-9, COX-2, IKK*α*, and IKK*β* in pancreatic tumor tissues. Samples from three animals in each group were analysed, and representative data are shown. *β*-Actin was used as loading control.

**Figure 4:**
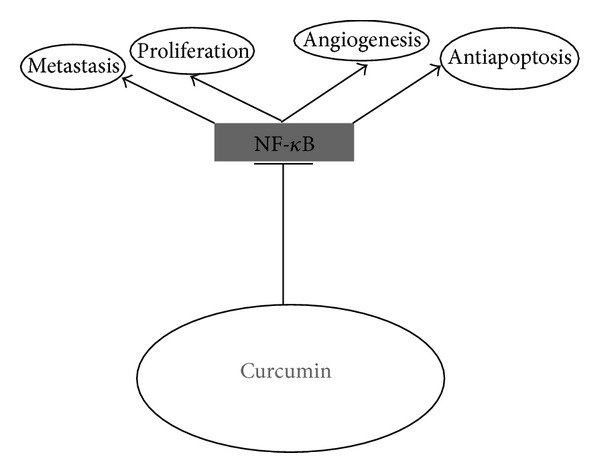
Molecular targets of curcumin in pancreatic cancer cells.

Since it has been demonstrated that curcumin potentiates the antitumor activity of gemcitabine in pancreatic cells by downregulating NF-*κ*B-regulated gene products [17], we performed DNA binding to detect NF-*κ*B expression in orthotopic tumor tissue samples from control and treated mice. Our results showed the inhibition of NF-*κ*B activation by curcumin (Figure 3(a), lane 2). Since it has been demonstrated that NF-*κ*B regulates the expression of several markers involved in proliferation (COX-2, cyclin D1), in invasion (MMP-9), and in angiogenesis (VEGF), we performed an immunohistochemical analysis and Western blotting on orthotopic tumor tissue samples from control and treated mice. Immunoistochemical analysis indicates that, in tumors of curcumin-treated group, there are significant reductions in the expression of COX-2 and VEGF, compared with the control group (Figure 3(b)). Western blotting analysis revealed that curcumin, significantly decreased the expression of all of these molecules compared with the control treatment in pancreatic tumor tissues (Figure 3(c)). We finally performed a western blot analysis with IKK*α* and IKK*β* in order to understand how curcumin inhibits NF-*κ*B activation in MIA PaCa-2 cells. Our data indicated that there is a reduced expression of IKK*α* and IKK*β* in tumor of mice treated with curcumin with respect to controls, indicating that curcumin inhibits NF-*κ*B activation through suppression of IKK (Figure 3(c)).  Figure 4 shows a schematic diagram which contextualizes the various signalling cascades affected by curcumin in pancreatic cancer cells. Altogether, these data indicate that curcumin inhibits NF-*κ*B activation and down-regulates NF-*κ*B-regulated gene products in orthotopic pancreatic tumors.

## 4. Discussion

Pancreatic cancer is a malignant neoplasm originating from transformed cells arising in tissues forming the pancreas. Gemcitabine is the best chemotherapeutic agent available for treatment of pancreatic cancer. However, it is noted that patients with this cancer treated with gemcitabine, showed several side effects and developed drug resistance over time [3]. In order to bypass these problems, novel strategies involving less toxic agents that can sensitize pancreatic cancer cells to chemotherapy, are necessary. Curcumin, a component of turmeric (*Curcuma longa*), is one such agent which inhibit the cell survival, proliferation, angiogenesis of a wide variety of tumor cells, through the modulation of various cell signalling pathways. It has been demonstrated that curcumin in different pancreatic cancer cell lines inhibited proliferation, potentiated the apoptosis induced by gemcitabine, and inhibited constitutive NF-*κ*B activation. Our aim was to determine whether curcumin, inhibits the growth and angiogenesis of MIA PaCa-2 human pancreatic cancer cells in *in vitro* and *in vivo* conditions. It has been largely demonstrated that curcumin is able to suppress the growth of several tumor cell lines, including drug-resistant lines. It suppresses the expression of cyclin D1, which is involved in progression of cell through cell cycle and is deregulated in many tumors [19]. Curcumin potentiates the apoptosis in tumor cells by activation of caspase enzymes and suppresses the activation of many transcription factors involved in tumorigenesis [20]. Recently it has been demonstrated that curcumin potentiates antitumor activity of gemcitabine in an orthotopic model of pancreatic cancer trough suppression of proliferation, angiogenesis, NF-*κ*B, and inhibition of NF-*κ*B-regulated gene products [17]. According to published data, we demonstrated, by *in vitro* experiments, that curcumin inhibits proliferation and enhances apoptosis in MIA PaCa-2 human pancreatic cancer cells. In addition to these *in vitro* results, we found that curcumin, administrated in mice with diet, inhibits tumor growth and angiogenesis in an orthotopic model of pancreatic cancer as indicated by a decrease in tumor volume of curcumin-treated mice, compared to controls. By EMSA assay, we demonstrated that curcumin suppressed constitutive NF-*κ*B activation in pancreatic cancer tissues according to previous data [17]. Our data also showed that curcumin inhibited the expression of several important proteins regulated by NF-*κ*B; in particular, we demonstrated that there is a reduced expression of IKK*α* and IKK*β* in tumor of mice treated with curcumin with respect to controls, indicating that curcumin inhibits NF-*κ*B activation through suppression of IKK in MIA PaCa-2 cells. Taken together, our results showed that that curcumin has antitumor effects in an orthotopic mouse model of human pancreatic cancer by inhibiting NF-*κ*B and its downstream targets. Since curcumin is a very well tolerated in human subjects and is assumed by food, our mouse model demonstrated curcumin can be used as an alternative agent to chemotherapy in treatment of human pancreatic cancer. Several *in vitro* and *in vivo* studies are on-going in our laboratory in order to test if a combination of curcumin (administrated by diet of loaded by nanoparticles) and gemcitabine can be used as possible and novel therapeutic schedule for pancreatic cancer.
